# Effect of different buried depth on the disintegration characteristic of red-bed soft rock and the evolution model of disintegration breakage under cyclic drying-wetting

**DOI:** 10.1038/s41598-024-57901-6

**Published:** 2024-04-01

**Authors:** Jun Zhang, Yang Guo, Kai Huang, Wei Cui, Zhaibang Ke, Xiaochuang Chen, Tengsheng Yue, Kun Gao

**Affiliations:** 1 Anhui Province Key Laboratory of Green Building and Assembly Construction, Anhui Institute of Building Research & Design, Hefei, 230601 Anhui China; 2 Anhui Construction Engineering Inspection Technology Group CO, LTD, Hefei, 230061 Anhui China; 3 College of Civil Engineering, Anhui Jianzhu University, Hefei, 230601 Anhui China; 4 Anhui Institute of Intelligent Underground Detection Technology, Anhui Jianzhu University, Hefei, 230601 Anhui China

**Keywords:** Red-bed soft rock, Disintegration characteristic, Drying-wetting cycles, Fractal theory, Energy dissipation, Environmental sciences, Hydrology, Natural hazards

## Abstract

The disintegration of red-bed soft rock exhibits a strong correlation with various geological disasters. However, the investigation into the evolutionary mechanisms underlying disintegration breakage has not yet received extensive exploration. In order to comprehensively examine the disintegration characteristics of red-bed soft rock, the slake durability tests were conducted to red-bed soft rocks of varying burial depths. Subsequently, an investigation was carried out to examine the disintegration characteristics and the evolution of disintegration parameters, including the coefficient of uniformity (*Cu*), coefficient of curvature (*Cc*), disintegration rate (*DRE*), disintegration ratio (*Dr*), and fractal dimension (*D*), throughout the disintegration process. Finally, employing the energy dissipation theory, an energy dissipation model was developed to predicate the disintegration process of samples at various burial depths. The findings demonstrate a decrease in the abundance of large particles and a concurrent increase in the abundance of small particles as the number of drying-wetting cycles increases. Furthermore, as the number of drying-wetting cycles increases, a significant alteration is observed in the content of particles larger than 10 mm, whereas the content of particles smaller than 10 mm undergoes only minor changes. The disintegration parameters, including the curvature coefficient, non-uniformity coefficient, disintegration rate, and fractal dimension, exhibit a positive correlation with the number of drying-wetting cycles. Conversely, the disintegration index demonstrates a decreasing trend with the increasing number of cycles. Nevertheless, as the burial depth increases, a notable trend emerges in the disintegration process, characterized by a gradual increase in the content of large particles alongside a progressive decrease in the content of small particles. Concurrently, the curvature coefficient, non-uniformity coefficient, disintegration rate, and fractal dimension exhibit a gradual decline, while the durability index experiences a gradual increase. In addition, based on the principle of energy dissipation, it is revealed that the surface energy increment of red-bed soft rock increases with the increase of the number of drying-wetting cycles, but decreases with the increase of burial depth. Ultimately, by leveraging the outcomes of energy dissipation analyses, a theoretical model is constructed to elucidate the correlation between surface energy and both the number of drying-wetting cycles and burial depth. This model serves as a theoretical reference for predicting the disintegration behavior of samples, offering valuable insights for future research endeavors.

## Introduction

Red-bed soft rock is widely distributed throughout China and represents a commonly encountered geological formation in various surface and subsurface engineering contexts^1,2^. Previous studies have illustrated the adverse engineering properties associated with red-bed soft rock, such as its notable expansibility and susceptibility to moisture, which may give rise to safety considerations^3–5^. Owing to its distinctive lithological attributes, the disintegration of red-bed soft rock is intricately associated with geological hazards and deformations, such as landslides and mudslides^6–9^. As a result, the disintegration phenomenon of red-bed soft rock has attracted considerable interest from researchers in the fields of engineering and geomorphology. Investigating the disintegration characteristics of red-bed soft rock carries substantial theoretical and practical implications.

In recent years, significant research endeavors have been directed towards the examination of red-bed soft rock disintegration through laboratory experiments. These investigations have been primarily focused on underlying disintegration mechanisms under hydrostatic conditions. Dick et al.^1^ noted the rapid deterioration observed in numerous soft rocks due to fluctuations in moisture content, a phenomenon they termed "slaking", which contributes significantly influence on slope instability and underground excavation. Moreover, the majority of prior researchers have utilized laboratory methodologies focusing on changes in water content to quantitatively evaluate the disintegration characteristics of soft rocks^10–16^. Among the widely recognized methodologies, the jar slake test^17^, the slake index test^18,19^, and the slake durability test^20,21^ stand out as the most frequently employed techniques in assessing the disintegration characteristics of soft rocks.

The disintegration of soft rock is a complex phenomenon influenced by multiple factors, and it has garnered significant attention from the research community. Zhao et al.^22^ conducted a comprehensive investigation by conducting disintegration tests on red sandstone under diverse conditions, encompassing water, atmospheric, and field environments. Based on the observed disintegration characteristics, they classified the red sandstone into three distinct types according to the degree of disintegration. Yan et al.^23^ undertook a comprehensive study delving into the disintegration dynamics of red-bed soft rock, emphasizing the impact of temperature difference (TD), wetting and drying (WD), and the synergistic effect of TD and WD (TDWD). Their findings revealed that the impact of TD on disintegration is particularly pronounced for soft rock samples with lower resistance to disintegration. According to recent studies, the cyclic drying and wetting process has emerged as a prominent determinant in the disintegration mechanism of red-bed soft rock^24–26^. Moreover, a number of scholars have introduced valuable disintegration indices^27–30^ aimed at characterizing the slaking behavior of rocks under cyclic wetting and drying (WD) conditions. Sadisun et al.^31^ and Zhou et al.^32^ underscored the diminishing trend observed in the durability index with escalating numbers of drying-wetting cycles. They additionally expounded upon the pivotal role of cyclic wetting and drying as a prominent mechanism driving the degradation and deterioration of rock materials.

The aforementioned achievements have successfully elucidated the disintegration properties of red bed soft rock under cyclic drying-wetting conditions. Nevertheless, it is imperative to acknowledge that during engineering construction, the collapse behavior of red-bed soft rock is impacted not solely by the drying and wetting cycles but also by fluctuations in burial depths. To elaborate, the softening degree of red-bed soft rock displays a gradient pattern in relation to burial depth, characterized by the most pronounced softening in shallow burial depths, followed by intermediate levels in the middle section, and the least degree of softening in deeper regions. Hence, the comprehensive consideration of both drying-wetting cycles and burial depth is essential for establishing a rational understanding of the collapse behavior of red-bed soft rock in practical engineering applications. However, the impact of drying and wetting cycles, as well as burial depth, on the disintegration of red-bed soft rock has received limited attention in prior literature. Therefore, this study investigated the influence of varying burial depths on the disintegration characteristics of red-bed soft rock under cyclic drying-wetting conditions. Subsequently, this study analyzes the disintegration characteristics and examines the disintegration parameters during the disintegration process. Finally, a theoretical model was developed to predicate the disintegration process of samples at various burial depths.

## Experiment

### Basic properties of samples

**Figure 1:**
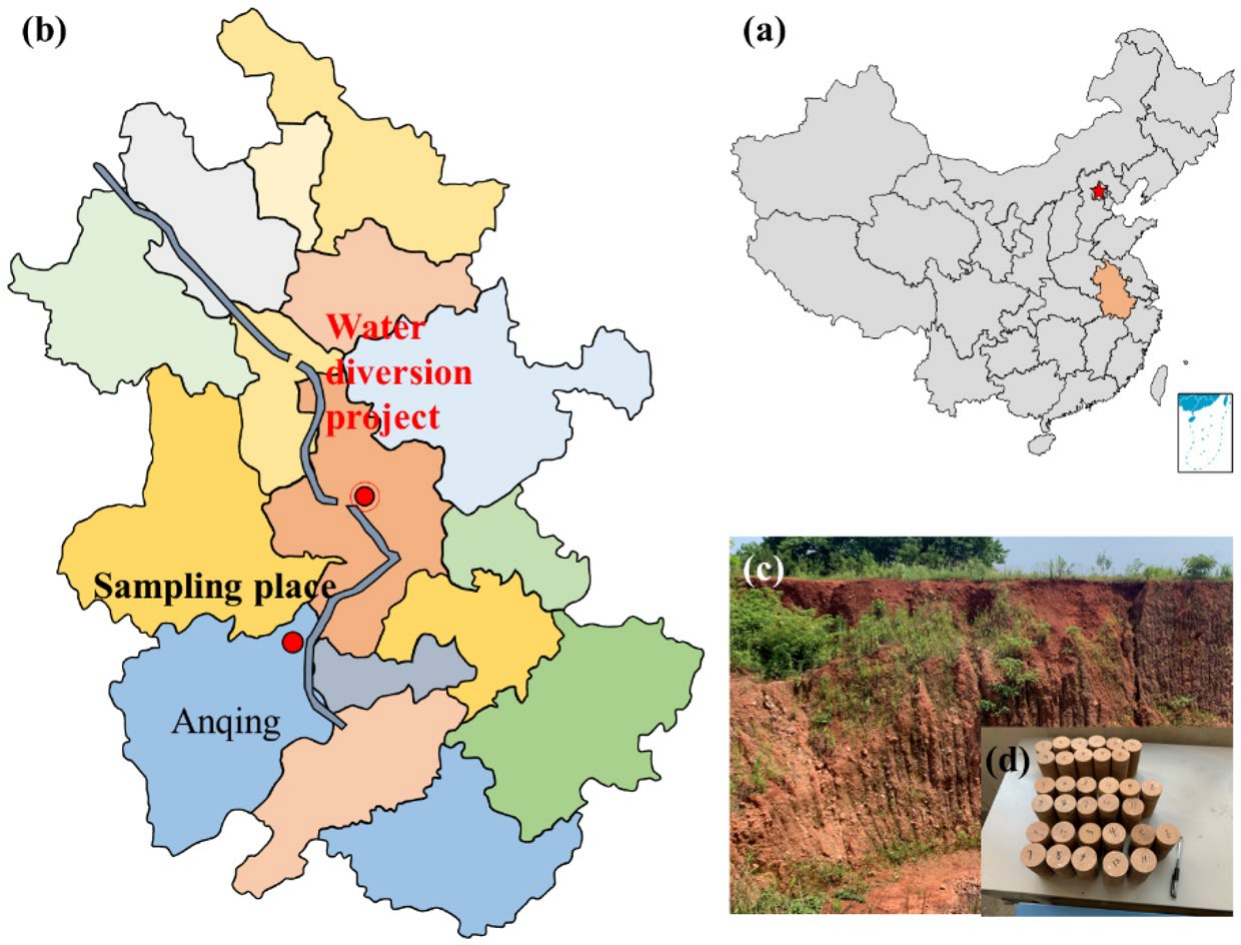
Sampling location of present study.

**Figure 2:**
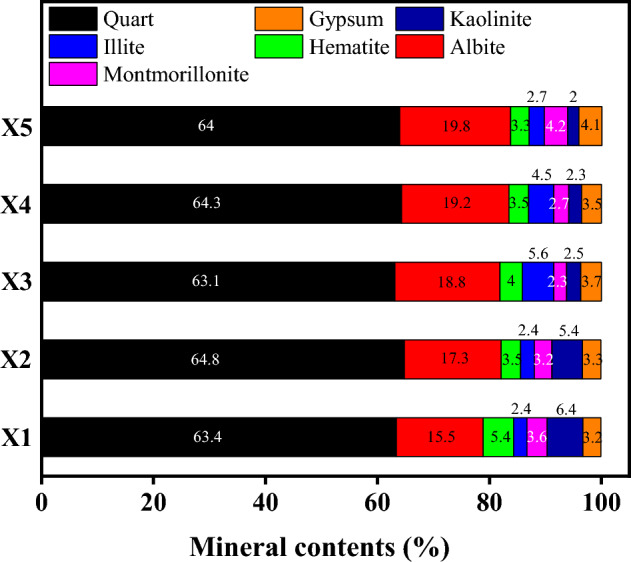
Evolution of mineral content of two kinds of red-bed soft rock with buried depth.

**Figure 3:**
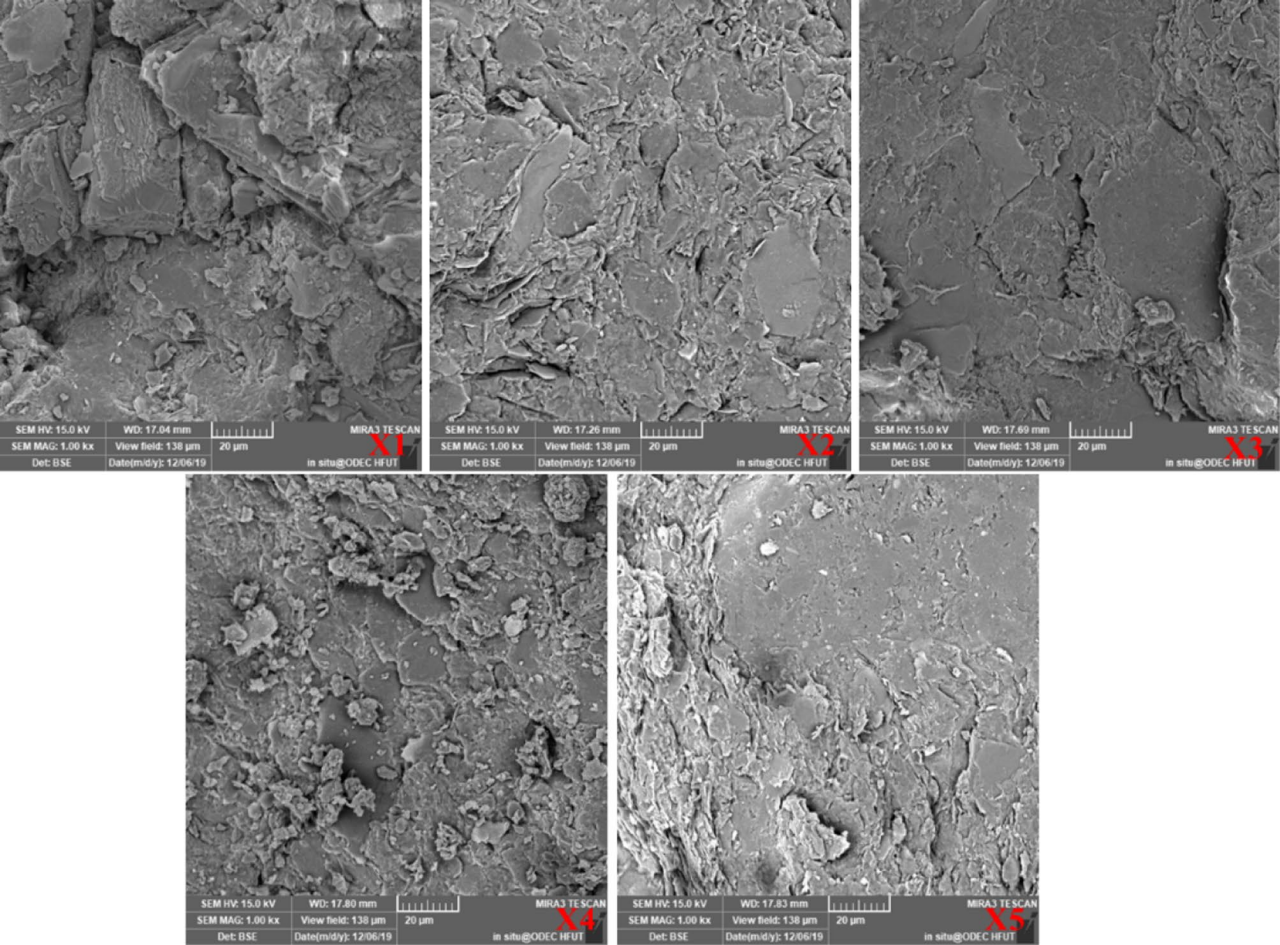
Microstructure images of tested samples.

The tested samples were red-bed argillaceous siltstone of the Huizhou Formation collected from Huangshan, Anhui Province, China (as shown in Fig. 1). To ensure homogeneity, the samples were collected from the same local area of the same site, and the sample number, sampling depth and the related physical properties of the samples were acquired and are shown in Table 1. X-ray diffraction (XRD) of the red-bed soft rock was conducted to acquire the mineral composition (as shown in Fig. 2). Figure 2 show that the main mineral component is quartz, and that the red-bed soft rock contains certain expansive clay minerals, such as montmorillonite, illite, and kaolinite. In addition, Electron microscopy was carried out on the soft rock to analyze its microscopic structure. Figure 3 shows images from a scanning electron microscope (SEM) of the red-bed soft rock at different sampling depth. The images show that the granular structure of the soft rock is relatively complete, and the pore distribution between particles is obvious. And with the increase of the depth, the microstructure is gradually dense and the porosity is obviously reduced.

**Table 1:** Sample number and sampling depth.

Lithology	Sample Number	Sampling depth (m)	Dry density (g/cm^3^)	Natural moisture content (%)	Porosity (%)
Argillaceous siltstone of Huizhou formation	X1	9.80–9.90	2.323	1.72	8.22
	X2	19.80–19.90	2.503	1.57	7.39
	X3	29.80–29.90	2.582	1.38	6.97
	X4	39.80–39.90	2.610	1.32	6.65
	X5	49.80–49.90	2.618	1.30	6.39

### Test procedures and methods

**Figure 4:**
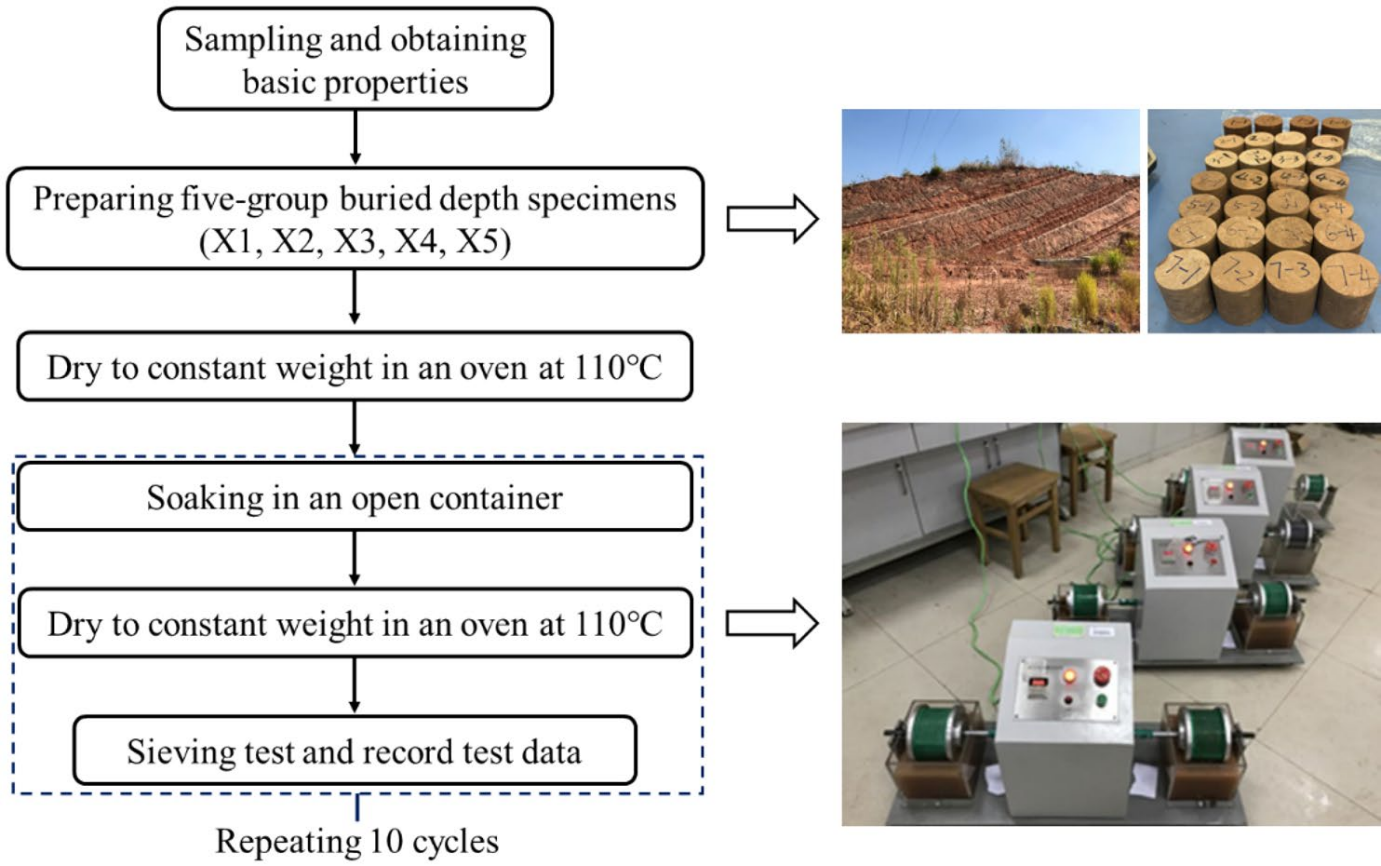
Test flow chart.

The disintegration experiment was the widely used experimental method to measure the durability of soft rock^32^. It was introduced by Franklin and Chandra^11^ and was later adopted as the ASTM standard method to test the durability of soft rock. In this paper, the test procedure of red-bed argillaceous siltstone is designed (as shown in Fig. 3), five groups of disintegration experiments were conducted, and the sample numbers are X1, X2, X3, X4, and X5. According to ASTM D4644-08^33^, the samples were cylinders with a diameter of 50 mm and a height of 100 mm (± 1 mm), and the initial mass of every rock sample was kept in the range of 0.39–0.41 kg. Samples of each group are shown in Fig. 4.

To eliminate the influence of the initial moisture content, each group of samples was dried before the test, and the experiment procedures suggestions by ASTM D5313-12^34^, Erguler and Ulusay^35^ and Gautam and Shakoor^28^; see Fig. 4 for test procedures. Specifically, (1) dry the natural samples in a 105 ℃ oven for 24 h to achieve thorough drying and then weigh them; (2) saturate the dried samples in deionized water for 24 h; (3) dry the samples in a 105 ℃ oven for more than 24 h and then cool the samples an ambient temperature (approximately 25 ℃, which was controlled in the laboratory) for 6 h; (4) sieve the disintegration products with mesh sizes of 40, 20, 10, 5, 2, 0.5, and 0.25 mm individually, and weigh the masses of grains with different mesh sizes. Furthermore, considering the operability of the drying-wetting cycles experiment combined with the ASTM D4644-16^36^, in this experiment, ten cycles of drying-wetting were carried out, and standard sieves were also used.

## Results

### Disintegration Phenomena

**Figure 5:**
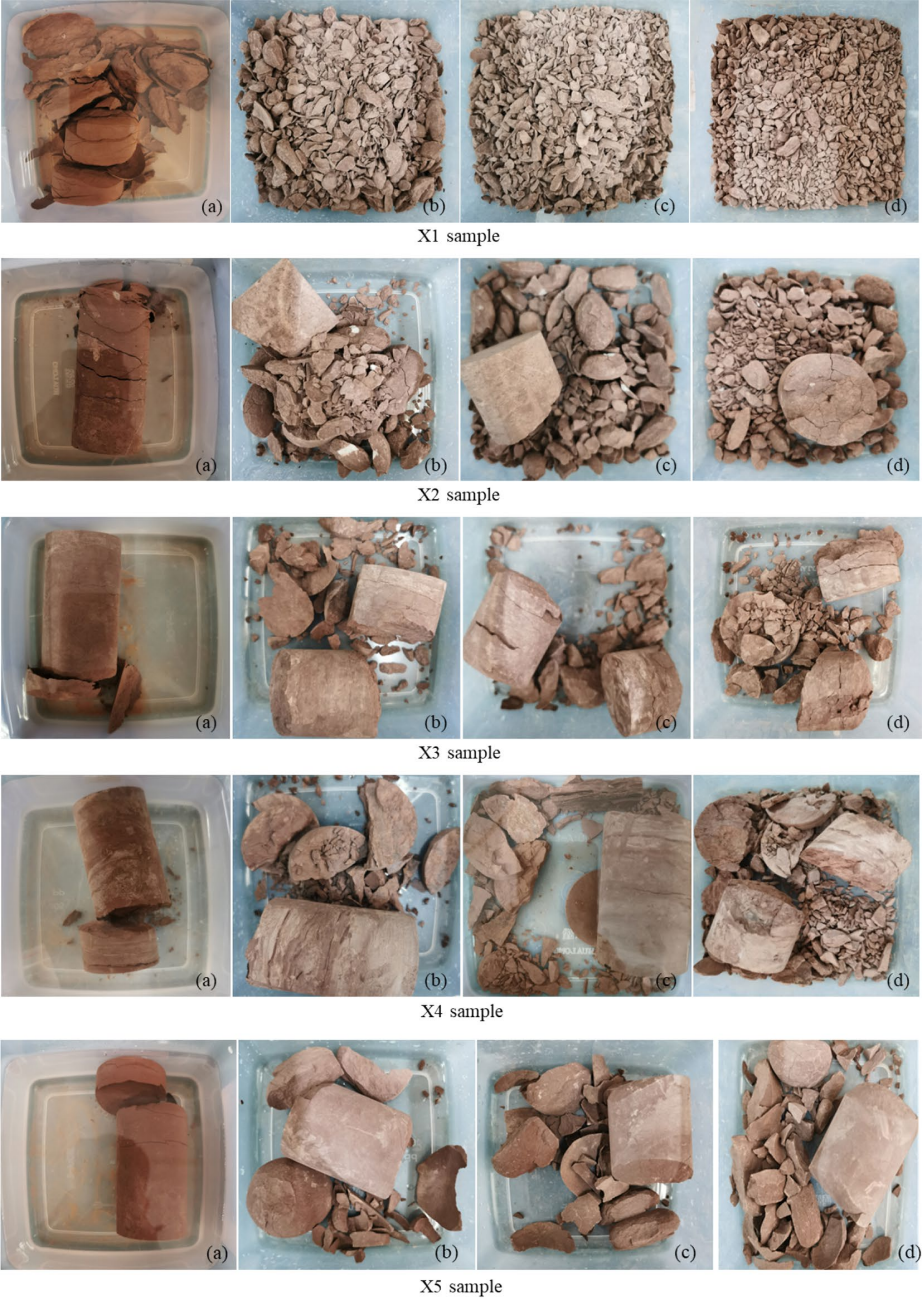
Disintegration of X1 sample: (**a**) cycle one; (**b**) cycle three; (**c**) cycle five; and (**d**) cycle ten.

The disintegration phenomena of red-bed argillaceous siltstone were investigated through the analysis of digital camera images. Based on these findings, the disintegration behavior of red-bed argillaceous siltstone under varying burial depths and cyclic drying-wetting conditions was elucidated, shedding light on its disintegration patterns. Photographs were captured for four sets of red-bed argillaceous siltstone samples, each corresponding to distinct burial depths. In consideration of spatial constraints, Fig. 5 exclusively depicts the morphological variations observed in the disintegrated X1 sample across different drying-wetting cycles. It is noteworthy that as the number of drying-wetting cycles increases, the red-bed argillaceous siltstone particles exhibit a trend towards decreased particle size. Over the initial three drying-wetting cycles, the red-bed argillaceous siltstone undergoes a progressive disintegration process, resulting in a morphological transformation characterized by the aggregation of gravel-like structures in the disintegrated material. Following the completion of five drying-wetting cycles, the presence of particles exceeding 20 mm in size becomes considerably scarce. Subsequently, between the fifth and tenth drying-wetting cycles, the red-bed argillaceous siltstone particles continue to experience progressive disintegration, albeit with minimal alterations in particle size distribution.

### Particle grading characteristics

**Figure 6:**
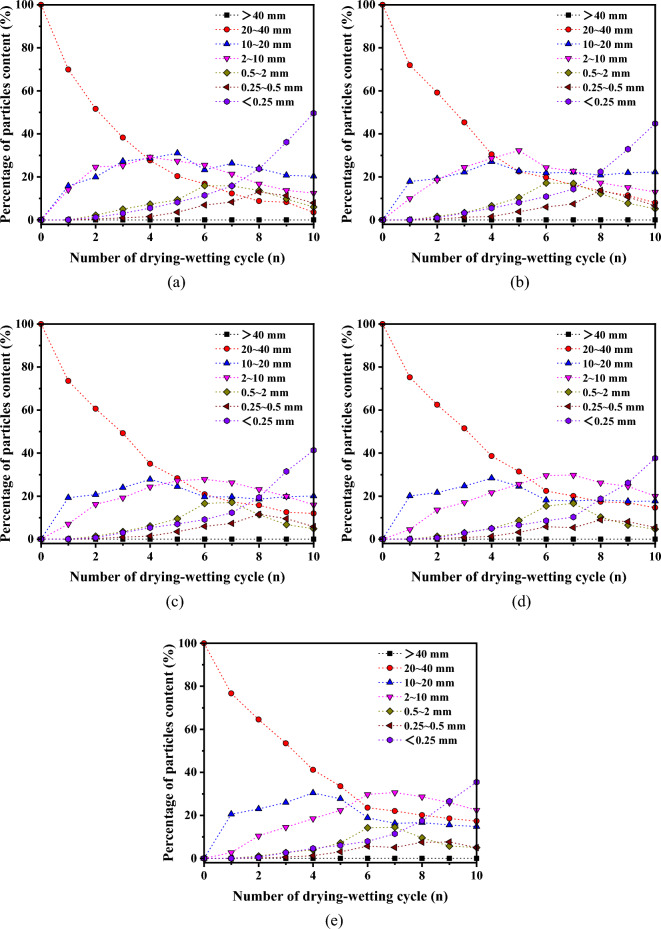
Disintegration characteristic curves of granules of samples during drying-wetting cycles. (**a**) X1 (**b**) X2 (**c**) X3 (**d**) X4 (**e**) X5.

The evolution of the grain content with various particle sizes as the number of drying-wetting cycles increased is illustrated in Fig. 6.

Figure 6 reveals a discernible trend in particle content variation as the number of drying-wetting cycles increases. To be specific, the particle content of particles ranging from 20–40 mm in size consistently diminishes, whereas the particle content of particles sized 10–20 mm undergoes an initial increment followed by a subsequent decrement, eventually stabilizing. The evolution trend of particle sizes spanning 2–10 mm, 0.5–2 mm, and 0.25–0.5 mm exhibits a similar pattern, characterized by an initial increase followed by a state of relative stability. In contrast, particles smaller than 0.25 mm demonstrate a persistent increase in size as the number of wetting and drying cycles rises. Furthermore, upon analyzing the disintegration characteristic curve, notable observations emerge. Firstly, sample X1 displays the highest content of particles smaller than 0.25 mm, whereas sample X5 exhibits the lowest content in this size range. Secondly, in terms of particles sized 20–40 mm, sample X1 records the lowest content, while sample X5 showcases the highest content. In conclusion, as the disintegration process progresses, there is a discernible trend in particle content with respect to occurrence depth. Specifically, there is a gradual increase in the content of larger particles, accompanied by a gradual decrease in the content of smaller particles.

## Discussions

### Analysis of disintegration parameters

**Figure 7:**
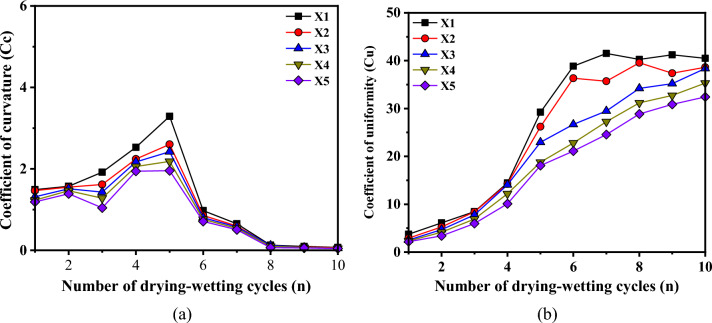
Evolution curves of Cc and Cu. (**a**) coefficient of curvature (**b**) Coefficient of uniformity.

Figure 7 illustrates the evolution characteristics of the curvature coefficient and non-uniformity coefficient pertaining to particle size in red-bed argillaceous siltstone throughout the drying-wetting cycle.

As depicted in Fig. 7, the curvature coefficient and unevenness coefficient of red-bed argillaceous siltstone at varying burial depths exhibit an initial rise followed by a gradual stabilization as the number of cyclic drying-wetting increases. This behavior can be attributed to the progressive reduction in particle size of disintegrated entities and the concomitant increase in the abundance of smaller disintegrated objects as the drying-wetting cycles multiply. Consequently, the curvature coefficient shows a corresponding gradual increment. Furthermore, the red-bed argillaceous siltstone exhibits a pronounced disintegration propensity, wherein the sample progressively breaks down into disintegrated objects of varying particle sizes as the number of drying-wetting cycles increases. Consequently, the unevenness degree of the sample intensifies, accompanied by a corresponding augmentation in the unevenness coefficient. Upon the complete disintegration of the sample, both the curvature coefficient and the inhomogeneity coefficient manifest a tendency towards stability. As the burial depth increases, there is an observed elevation in the internal integrity of the sample, accompanied by a progressive enhancement in the inter-particle cohesion. This phenomenon leads to a deceleration in the expansion of fractures and a reduction in the rate of sample disintegration during the collapse process. Consequently, as the burial depth increases, there is a gradual decrease observed in both the curvature coefficient and the inhomogeneity coefficient.

### Disintegration ratio and durability index

#### Disintegration ratio

Given the low susceptibility of minerals like quartz and mica to disintegrate, it is observed that during the disintegration process, despite the stabilization of particles with sizes exceeding 2 mm, there persist certain substances that exhibit resistance to disintegration. Hence, the Disintegration Rate Efficiency (DRE) of red bedded argillaceous siltstone can be determined through the following calculation.

1 $$DRE = \frac{{m_{n1} }}{{m_{n0} }}$$

where, *m*_*n*0_ is the total mass of the sample after *n* drying-wetting cycles, and *m*_*n*1_ is the mass of disintegrant with particle size less than 2 mm after *n* drying-wetting cycles.

**Figure 8:**
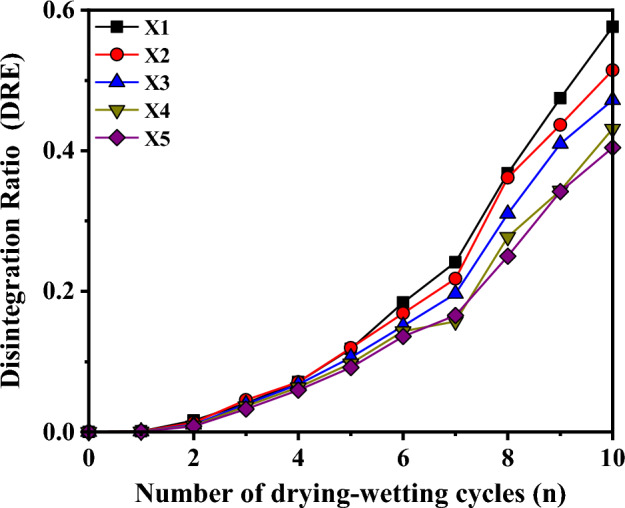
Evolution curve of *DRE.*

The disintegration rate evolution curve obtained is depicted in Fig. 8, wherein it is evident that an incremental trend in the disintegration rate is observed with the progressive increment in the number of drying-wetting cycles. This can be attributed to the intensified microstructural damage of the red bed argillaceous siltstone, as a result of increased drying-wetting cycles, leading to the complete dissolution of clay minerals and exacerbating the disintegration process within the sample. Furthermore, during the initial five drying-wetting cycles, the disparity in the disintegration rate among samples at various burial depths is minimal. However, as the number of drying-wetting cycles increases, the distinction in the disintegration rate becomes progressively pronounced. Notably, a gradual reduction in the disintegration rate is observed with the augmentation of the occurrence depth. This observation suggests that as the occurrence depth increases, the susceptibility to disintegration of the sample gradually diminishes, while concurrently enhancing the resistance to disintegration.

#### Durability index

In previous investigations, the evaluation of soft rock disintegration characteristics has commonly relied on the durability index derived from two drying-wetting cycle tests. However, this approach exhibits certain limitations. Consequently, in light of these considerations, the present study proposes a modification to the durability index (*I*_*dn*_), which is as follows:

2 $$I_{dn} = \frac{{m_{n} }}{{m_{0} }} \times 100\%$$

where, *I*_*dn*_ is the durability index after the nth cycle, *m*_0_ is the initial dry weight of the sample, (g), and *m*_n_ is the weight of the retained portion of the samples after the nth cycle, (g).

**Figure 9:**
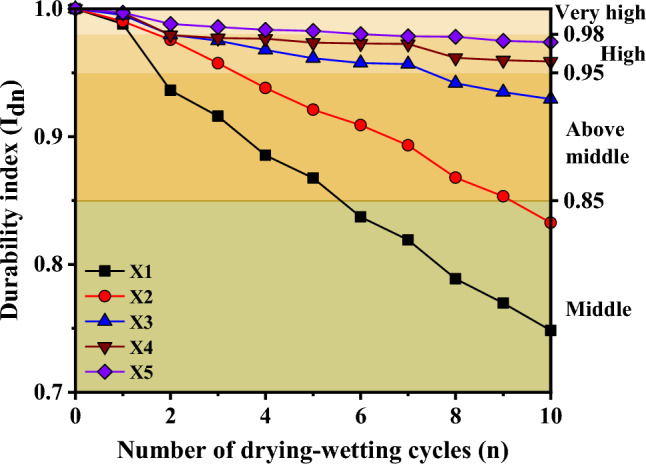
Exponential curve of disintegration resistance.

As depicted in Fig. 9, it is evident that the durability index of the sample gradually decreases with an increasing number of drying-wetting cycles. This observation implies that as the number of drying-wetting cycles rises, the durability of sample gradually strengthens, while its ability to withstand disintegration diminishes progressively. With the increase of burial depth, the durability index of the sample gradually increases, which is consistent with the results obtained in the previous paper, because with the increase of the occurrence depth, the pore structure gradually densifies and the clay mineral content gradually decreases.

### Fractal model of disintegration

#### Fractal theory

The disintegration results of red bed argillaceous siltstone show that the macroscopic fracture of the sample is caused by the formation, expansion and aggregation of internal micropores. The disintegration of red-bed argillaceous siltstone after water absorption is random and its failure process is also a fractal process. Therefore, it is feasible to use the fractal theory to investigate the disintegration mechanism of rocks.

Fractal theory encompasses the examination of irregular shapes and curves characterized by self-similarity, employing fractal dimensions to quantify the distribution of fractions^37,38^. Turcotte^38^ identified various calculation methods utilized to determine the fractal dimension, namely the box-counting method, self-similar dimension method, and scale-free correlation dimension method. Based on the fundamental principles of fractal theory, the present study integrates the unique characteristics of disintegration in red-bedded argillaceous siltstone and employs fractal dimension and particle size as key parameters for determining the fractal dimension. It is imperative that the relationship between particle size and fractal dimension adheres to Eq. (3).

3 $${N}_{\left(r>R\right)}\propto {R}^{-D}$$

where *N*_*(r*>*R)*_ is the number of particles with a size greater than R, *D* is the number of fractal dimensions.

Generally, the distribution of disintegration product sizes can be described by the Weibull distribution, which is widely applicable in characterizing the size-frequency relationship, namely:

4 $$\frac{{M\left( {r \le R} \right)}}{M} = 1 - e^{{\left[ { - \left( {\frac{R}{{R_{T} }}} \right)^{\theta } } \right]}}$$

where *M*_*(r*>*R)*_ is the cumulative mass of the sample with a particle diameter less than R, *M* is the total mass of samples after disintegration, *R*_*T*_ is average radius of particles after disintegration, and *θ* is the exponent.

Expanding formula (4) as a Taylor series while neglecting higher-order terms leads to the derivation of the following equation.

5 $$e^{{\left[ { - \left( {\frac{R}{{R_{T} }}} \right)^{\theta } } \right]}} = 1 - \left( {\frac{R}{{R_{T} }}} \right)^{\theta }$$

From Eqs. (4),(5), the following formula can be obtained.

6 $$\frac{{M_{{\left( {r < R} \right)}} }}{M} = \left( {\frac{R}{{R_{T} }}} \right)^{\theta }$$

Furthermore, according to Eq. (5) and Eq. (4), *N∝R*^*D-1*^${\text{dR}}$ and dM*∝R*^*θ-1*^${\text{dR}}$ can be obtained, respectively. Hence:

7 $$R^{ - D - 1} dR \propto R^{\theta - 4} dR$$

Finally, the fractal dimension *D* can be expressed by:

8 $$D = 3 - \theta$$

Equation (8) is substituted into Eq. (6) to obtain the mass-based form of the fractal model (Zeng et al. 2021). It is expressed as follows:

9 $$\frac{{M_{{\left( {r < R} \right)}} }}{M} = \left( {\frac{R}{{R_{T} }}} \right)^{3 - D}$$

#### Fractal analysis of the red-bed soft rock

**Figure 10:**
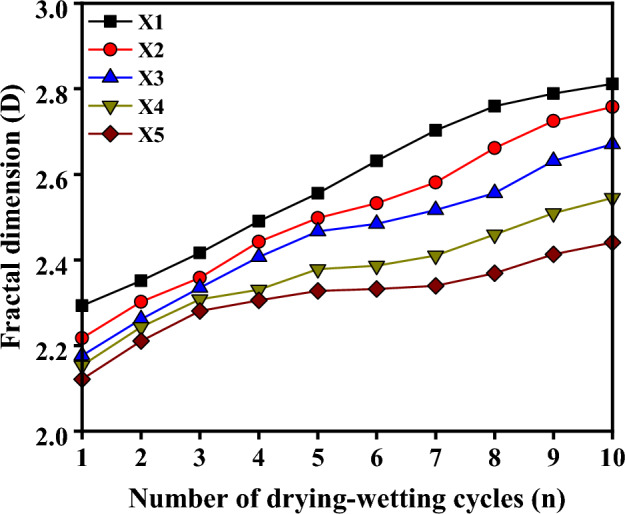
Evolution curves of fractal dimension of red-bed soft rock under cyclic drying-wetting.

Figure 10 illustrates the determination of the fractal dimension *D* for red-bed argillaceous siltstones at various buried depths subjected to drying-wetting cycling, based on the calculations derived from the aforementioned formula.

From the observations depicted in Fig. 10, it is evident that the fractal dimension of the samples at varying burial depths exhibited a gradual increase throughout the initial six drying-wetting cycles, followed by a subsequent stabilization, which is consistent with the variation of the aforementioned disintegration degree of the red-bed argillaceous siltstones. The dynamic changes in the fractal dimension offer valuable insights into the disintegration rate of the sample. Notably, under identical drying-wetting cycles, there exists an inverse correlation between the fractal dimension and the depth parameter. Consequently, an increase in depth corresponds to a gradual decrease in the fractal dimension. This observation indicates a reduction in the proportion of coarse particles and an augmentation in the abundance of fine particles within the argillaceous siltstone as the burial depth intensifies. Furthermore, a smaller disintegration rate aligns with a heightened resistance to disintegration, supporting the findings reported in the preceding study. The aforementioned findings demonstrate that the parameter D serves as an effective indicator of the disintegration extent of red-bed argillaceous siltstone throughout the disintegration process, rendering it a valuable tool for informing engineering applications and practices.

### Energy dissipation analysis of red-bed soft rock

The findings indicate that the disintegration mechanism of red-layer argillaceous siltstone involves the dissipation of energy^39^. During the disintegration process, the dissipated energy primarily originates from the heat absorbed through the evaporation of water loss from the sample. During the process of sample disintegration, the dissipation of energy primarily manifests in the forms of elastic deformation energy, plastic deformation energy, and surface energy^29^.

Due to the presence of cracks and micro-cracks in the structure of soft rock, it possesses a significant surface energy, facilitating water absorption upon contact. Moreover, the surface energy of these cracks and micro-cracks gradually diminishes, leading to the formation of a surface adsorption layer. A substantial portion of the reduced surface energy undergoes conversion into mechanical failure energy, thereby contributing to the expansion of the rock phase surface. The significance of surface energy in energy transfer and conversion is evident. While the conversion of surface energy is relatively straightforward, other energy conversion processes in the system exhibit greater complexity. Hence, this study aims to investigate the evolution of surface energy during the disintegration of red-bed argillaceous siltstone, with the objective of revealing the disintegration characteristics of red-bed argillaceous siltstone at varying burial depths.

Assuming an initial particle size of *R* prior to calving, the corresponding initial surface area can be estimated as 4π*R*^2^. After the sample has disintegrated into particles with radius *r*_*i*_, the total surface area is $\sum_{i=1}^{n}4\pi {r}_{i}^{2}$. According to the sieve test method^40^, the approximate total surface area can be expressed as:

10 $$A = \sum\limits_{i = 1}^{N} {\frac{{4\pi R^{3} P_{i} }}{{\overline{r}_{i} }}}$$

where, *P*_*i*_ is the mass percentage of particles in any particle size range, $\overline{{r}_{i}}$ Is the average radius of its corresponding particle size range.

Thus, the increment in the surface area after disintegration can be calculated as:

11 $$\Delta A = 4\pi R^{2} \left( {\sum\limits_{i = 1}^{N} {\frac{{4\pi RP_{i} }}{{\overline{r}_{i} }} - 1} } \right)$$

Furthermore, the unit surface energy of rock, as proposed by Zhao^41^, can be mathematically represented as follows:

12 $$G_{1C} \left( r \right) = \frac{{\pi \sigma_{R}^{2} L^{{d_{f} }} \left( {G\gamma V} \right)^{d} }}{{\alpha^{2} \Gamma^{2} \left( {1 + \frac{1}{2d}} \right)\left( {1 - D} \right)^{{2\lg \left( {R/r} \right)}} E}}$$

where, *α* represents the stress state factor, which is assumed to have a value of 1; *σ*_*R*_ denotes the tensile strength of the sample with a radius of R; *L* corresponds to the grain size of the constituent rock; *d*_*f*_ is the fractal dimension of crack distribution; *G* represents the constant related to the load system and crack orientation; *d* represents the fractal dimension of crack distribution; *γ* signifies the average density of cracks; *V* denotes the volume of the sample; *r* represents the particle radius following sample collapse; *Γ* refers to the Gamma function; *D* represents the damage variable; *E* represents the elasticity modulus.

In this paper, it is assumed that the parameter α and elastic modulus *E* of the red shale siltstone at different buries depths are invariants during the disintegration process, Additionally, assumed the ${\text{S}}={L}^{{d}_{f}}{\left(G\gamma V\right)}^{d}/{\Gamma }^{2}\left(1+1/2d\right)$ as an invariable factor. Based on these assumptions, the new surface energy after the first drying-wetting cycle as follows:

13 $$\Delta W = \frac{{4\pi R^{2} \sigma_{R}^{2} S}}{{\alpha^{2} E}}\left( {\sum\limits_{i = 1}^{N} {\frac{{4\pi RP_{i} }}{{\overline{r}_{i} \left( {1 - D} \right)^{{2\lg \left( {R/r} \right)}} }} - 1} } \right)$$

Upon completing a series of n drying-wetting cycles, the incremental surface energy can be derived through the following formulation:

14 $$\Delta W_{j} = \sum\limits_{i = 1}^{N} {\frac{{4\pi^{2} R^{3} \sigma_{R}^{2} S}}{{\alpha^{2} E\overline{r}_{i} \left( {1 - D} \right)^{{2\lg \left( {R/r} \right)}} }}} \left( {P_{i,j} - P_{i,j - 1} } \right)$$

where, *j* is the number of drying-wetting cycles.

**Figure 11:**
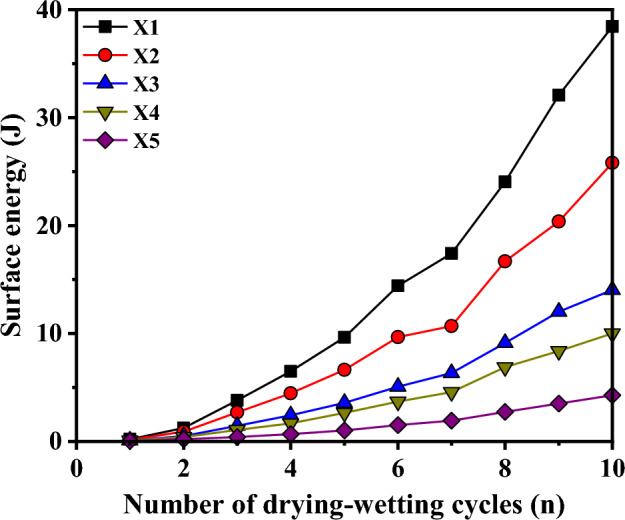
Evolution curves of surface energy of red-bed soft rock under cyclic drying-wetting.

As previously stated and in accordance with the experimental findings and literature references, the surface energy of the red-bed argillaceous siltstone was evaluated using Eq. (15). The variation of surface energy with respect to the number of drying-wetting cycles is illustrated in Fig. 11.

As depicted in Fig. 11, it is evident that for a given number of drying-wetting cycle, the incremental surface energy of the red-bed argillaceous siltstone diminishes as the buries depth increases. This observation is particularly pronounced after the fourth drying-wetting cycle, where the divergence in the incremental surface energy among the samples becomes progressively noticeable. After undergoing ten cycles of drying-wetting conditions, the surface energy of sample X1 exhibited a substantial increase of 38.44 J, in stark contrast to sample X5 which experienced a relatively modest rise of only 4.28 J. These results clearly demonstrate the varying degrees of disintegration, with sample X5 exhibiting weaker disintegration compared to the notably robust disintegration observed in sample X1. It is evident that the disintegration of the sample gradually diminishes as the buries depth increases. Furthermore, the surface energy increment of red-bed argillaceous siltstone exhibits a gradual rise with an increasing number of drying-wetting cycles, albeit with noticeable variations observed in the surface energy increments among individual samples. Among these, the surface energy increment of the two sample groups with shallow buries depth, namely X1 and X2 samples, exhibited a rapid increase after the second drying-wetting cycle. Conversely, for X3, X4, and X5 samples, the incremental change of surface energy was relatively slow during the initial four drying-wetting cycles, gradually approaching stability after eight cycles. This observation further supports the notion that samples with deeper buries depths experience weaker disintegration during the drying-wetting cycles.

Through further analysis of the surface energy increment during the drying-wetting cycle of red-bed argillaceous siltstone, the relationship between the surface energy increment and the number of drying-wetting cycles is obtained, as presented below:

15 $$\Delta W_{j} = a + be^{cx}$$

where: *n* is the number of drying-wetting cycles, and *a*, *b* and *c* are the model coefficients respectively.

**Figure 12:**
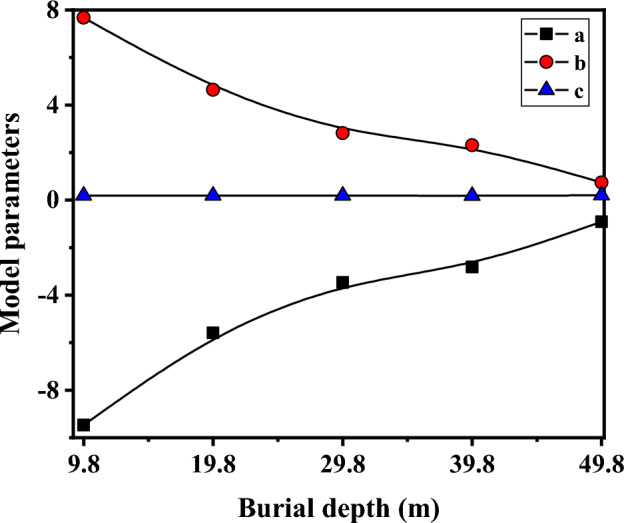
Evolution curve of model parameters with burial depth.

Utilizing the aforementioned equations, the surface energy of red-bed argillaceous siltstone at various buries depths was individually fitted. The outcomes, presented in Table 2, demonstrate fitting coefficients exceeding 0.991 for all cases, indicating a favorable fitting performance. Through a comprehensive analysis of the model parameters a, b, and c, as presented in Table 2, notable observations emerge regarding their correlation with burial depth. Specifically, it is observed that parameter a exhibits a gradual decrease, while parameter b displays a progressive increase as the burial depth increases. Furthermore, the value of parameter c remains relatively constant, hovering around approximately 0.18. These findings are visually depicted in Fig. 12.

**Table 2:** Model parameters calculation results of red-bed soft rock at different burial depths.

Sample number	Model parameters			Fitting coefficient
	a	b	c	
X1	− 9.47	7.67	0.183	0.997
X2	− 5.59	4.64	0.191	0.993
X3	− 3.47	2.82	0.184	0.994
X4	− 2.82	2.31	0.173	0.991
X5	− 0.91	0.74	0.196	0.992

## Conclusions

From the disintegration experiment on the red-bed argillaceous siltstone under cyclic drying-wetting, the following conclusions can be drawn:

- (1) As drying-wetting cycles increase, noticeable changes occur in particles larger than 10 mm, while the evolution of particles smaller than 10 mm shows a more gradual transition. Additionally, increasing occurrence depth during disintegration leads to a rise in large particles and a decrease in small particles.
- (2) The curvature coefficient, non-uniformity coefficient, and disintegration rate of red-bed argillaceous siltstone positively correlated with the number of drying-wetting cycles, irrespective of occurrence depth. However, an intriguing trend emerged with increasing depth, where internal integrity improved, leading to enhanced particle cohesion. Consequently, the curvature coefficient, non-uniformity coefficient, and disintegration rate gradually decreased, indicating higher disintegration resistance.
- (3) The disintegration process of red-bed argillaceous siltstone displays notable fractal characteristics, with a significant initial increase in fractal dimension over the first six drying-wetting cycles, followed by stabilization. Moreover, consistent drying-wetting cycles resulted in a gradual decrease in fractal dimension with increasing occurrence depth.
- (4) This study investigates energy dissipation characteristics and surface energy increment variations during drying-wetting cycles, providing insights into red-bed argillaceous siltstone calving. Additionally, it develops a predictive model for energy dissipation during disintegration, offering theoretical guidance for predicting sample dynamics.

## Data Availability

The data that support the findings of this study are available on request from the corresponding author.
